# Wernicke's encephalopathy due to malnutrition and parenteral nutrition in a patient with cerebral infarction: A case report

**DOI:** 10.1097/MD.0000000000019799

**Published:** 2020-04-17

**Authors:** Xiaojiao Lian, Meng Wu, Haixia Fan, Yi Zhang, Ping Sun

**Affiliations:** a Department of Nutrition, the First Hospital of Shanxi Medical University; b Department of Nutrition and Food Hygiene, School of Public Health, Shanxi Medical University; c Department of Geriatrics, the First Hospital of Shanxi Medical University, Taiyuan, Shanxi, China.

**Keywords:** cerebral infarction, malnutrition, parenteral nutrition, thiamine, wernicke's encephalopathy

## Abstract

**Introduction:**

Wernicke's encephalopathy (WE) is a severe neuropsychiatric disorder, which results from a nutritional deficiency of thiamine. The occurrence of WE is rarely reported in patients with cerebral infarction, who often have complications of malnutrition. Cerebral infarction is a neurological disease, patients with cerebral infarction may show symptoms such as disturbance of consciousness and gait instability, which is difficult to differentiate from WE. Thus, early recognition and differential diagnosis of WE are important. We report a rare case of cerebral infarction patient who developed WE due to malnutrition and parenteral nutrition.

**Patient concerns:**

A 65-year-old woman was admitted to our hospital with cerebral infarction. She had lost 15 kg of weight in the past month or so and was diagnosed with malnutrition. In order to correct malnutrition, parenteral nutrition and intravenous glucose without thiamine were administered. Cognitive dysfunction, laloplegia, sleep rhythm inversion, somnolence and bilateral lower limbs weakness were presented 20 days after admission.

**Diagnosis:**

Brain magnetic resonance imaging confirmed the diagnosis of WE.

**Interventions:**

The patient was given thiamine and nutrition support therapy.

**Outcomes:**

The patient's cognitive impairment, laloplegia and sleep condition improved within 4 days. Neurological status continued to improve and physical activity recovered gradually within 2 weeks. She received rehabilitation training when her condition was relatively stable, and her muscle strength of limbs and physical function gradually improved.

**Conclusion:**

Infarction-related malnutrition may result in nutrient deficiency-related neurological complications, such as WE. Thus, it is important to pay close attention to the nutritional status of patients with cerebral infarction. In addition, early recognition and differential diagnosis of WE in patients with infarction-related malnutrition are necessary, early treatment of replete thiamine supplementation and nutrition support therapy can reduce the risk of WE and improve the prognosis.

## 1 Introduction

Wernicke's encephalopathy (WE), first described by Carl Wernicke in 1881, is a severe neuropsychiatric disorder due to a nutritional deficiency of thiamine (vitamin B_1_).^[1]^ WE is characterized by a typical triad of altered mental status, ataxia of gait and ocular sign.^[2]^ Only 16% of WE patients showed the typical triad syndrome and 19% had no documented clinical signs.^[3]^ Although the current level of diagnosis and treatment has improved, there is still a potential underdiagnosis of WE.

WE is commonly caused by chronic alcoholism. Besides, WE also frequently occurs in people with fasting, starvation, malnutrition, malignant disease, gastrointestinal disease, and surgery,^[4]^ bariatric surgery,^[5]^ and hyperemesis gravidarum.^[6]^ Because stroke can affect the nutritional status of patients, malnutrition is often observed in such patients,^[7]^ which is a risk factor for WE. Cerebral infarction, a neurological disease accompanied with symptoms of disturbance of consciousness and gait instability, is difficult to differentiate from WE. However, WE is rarely reported in patients with cerebral infarction.

Here, we report a case of WE due to malnutrition and parenteral nutrition in a patient with cerebral infarction.

## 2 Case report

A 65-year-old woman showed weakness of the left limbs, lethargy and confusion with no obvious incentive on November 13th, 2017, so she visited a primary hospital and was diagnosed with cerebral infarction by brain magnetic resonance imaging (MRI). She experienced nausea and vomiting intermittently, even developed into poor appetite. Then intravenous hydration was administered and the specific condition was not clear, but the treatment produced little effect. Finally, the patient hospitalized in the Department of Geriatrics of our hospital on December 20th, 2017. At the time of admission, the height of the patient was 158 cm, the weight was 50.0 kg and the BMI was 20.0 kg/m^2^. Her weight had fallen by 15 kg for about a month. The NRS2002 nutritional risk screening tool scored 5 points.^[8]^ She was diagnosed with malnutrition. She suffered from hypertension for years and took levamlodipine besylate tablets orally. She also had a history of type 2 diabetes for more than one year and took metformin hydrochloride tablets orally. In addition, the patient had no history of smoking or alcoholism.

The patient cooperated well with the physical examination on admission. She manifested as the decline in numeracy and comprehension, right-left disorientation, tiny right horizontal nystagmus. The left finger-to-nose test was positive, and the heel-knee-tibia test could not be completed. Abdominal ultrasonography revealed gallbladder enlargement and the possibility of cholecystitis with cholestasis. Computerized tomography of the upper abdomen also showed gallbladder enlargement and cholestasis. Cardiac ultrasound revealed mild left atrial enlargement, mild mitral incompetence, and aortic sclerosis. Carotid artery ultrasound indicated bilateral carotid atherosclerosis with plaque formation. Initial laboratory findings were as follows: white blood cell count 6.4∗10^9^/L, elevated erythrocyte (5.66∗10^12^/L), hemoglobin level 145.0 g/L, platelet count 172∗10^9^/L, alanine aminotransferase level 21U/L, aspartate aminotransferase level 22U/L, elevated total bilirubin (36.4 μmol/L), elevated urea nitrogen (9.49mmol/L), creatinine level 61.3 μmol/L, hypokalemia (3.23mmol/L), serum sodium level (137mmol/L), hypochloremia (96.0mmol/L). The patient received intravenous hydration and symptomatic treatment. She received ondansetron to improve nausea and vomiting, with no significant improvement of symptoms. Considering the patient's disease status and nutritional status, the patient was finally given central venous nutrition. Because clinicians lacked systematic cognition of nutrition therapy, the central venous nutrition given to the patient only contained 50% glucose injection, compound amino acid injection (18AA-II), and 20% long chain fat emulsion injection, which made the nutrition imbalanced and vitamin deficient.

**Figure 1:**
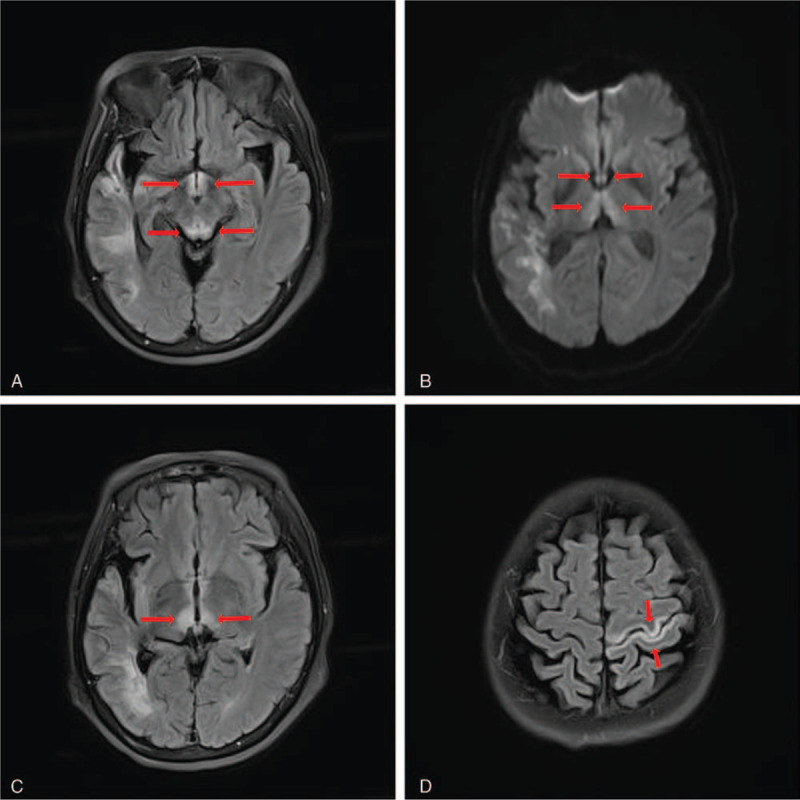
A. Fluid-attenuated inversion-recovery coronal images showing hyperintense signal alteration at the level of the periaqueductal gray matter and at the level of the mamillary bodies. B. Diffusion-weighted imaging images showing hyperintense signal alteration at the level of the mamillary bodies and at the level of the dorsal thalamus. C. Fluid-attenuated inversion-recovery coronal images showing hyperintense signal alteration at the level of the dorsal thalamus. D. Fluid-attenuated inversion-recovery coronal images showing hyperintense signal alteration at the level of the left central anteroposterior cortex.

On January 9th, 2018, the patient was critically ill and suddenly showed cognitive dysfunction, laloplegia, sleep rhythm inversion and then developed into somnolence. Bilateral lower limbs weakness and reduced activity were presented. The muscle strength of the right limbs was at the 1 level, the muscle tone was reduced. The heel-knee-tibia test could not be completed and bilateral babinski signs were positive. Therefore, the suspicion of a recurrence of cerebral infarction or the occurrence of a new lesion could not be ruled out. Acute laboratory results revealed low serum sodium (127mmol/L, normal range: 137–147mmol/L) and low serum chlorine (96mmol/L, normal range: 99–110mmol/L) levels, so hyponatremia and hypochloraemia should also be considered to be involved in altered mental status. Finally, MRI of the brain revealed abnormal signal in the dorsal thalamus, mamillary bodies, periaqueductal gray matter, left central anterioposterior cortex (Fig. 1), which was consistent with the diagnosis of WE.^[4]^

After confirmed the diagnosis, the patient received 100 mg of thiamine intramuscularly 3 times daily for 9 days and then 100 mg of thiamine intramuscularly two times daily for 4 days. At the same time, the patient asked for consultation of Department of Nutrition. We firstly recommended a gradual transition from parenteral nutrition to enteral nutrition to restore her intestinal function gradually. Considering that she had dysphagia and poor food intake due to cerebral infarction, we suggested to use an indwelling nasogastric tube. Initially, she was given partial parenteral nutrition and partial enteral nutrition for 6 days. That is 250 mL of compound amino acids (18AA-II) was administered once daily in partial parenteral nutrition. As for partial enteral nutrition, the intacted protein-based enteral nutrition powder was prepared into a nutrition solution with a concentration of 20% to 25%, and pumped into the nasogastric tube at a constant speed. Then, according to the patient's gastrointestinal tolerance, she gradually transitioned to total enteral nutrition, that is, the compound amino acid injection (18AA-II) was replaced by whey protein powder. In the first 3 days of the start of nutrition therapy, she was given 900 kcal of energy and 50 g of protein. Then, the energy and protein supplements were increased to 1300 kcal and 70 g, respectively. In addition, considering her long-term poor diet, she was given water-soluble vitamins, fat-soluble vitamins and trace elements supplements in enteral nutrition. Interestingly, after regular thiamine and nutritional treatment, her cognitive impairment, laloplegia and sleep condition significantly improved within 4 days. Her neurological status continued to improve and physical activity gradually recovered within 2 weeks. Subsequently, the patient, whose condition was relatively stable, was given 10 mg of thiamine orally three times daily and transitioned from enteral nutrition to food homogenate (low-salt and low-fat diabetes homogenate). As expected, her nutrition-related laboratory indicators and electrolytes gradually returned to normal level. Laboratory data are shown in Table 1.

**Table 1:**
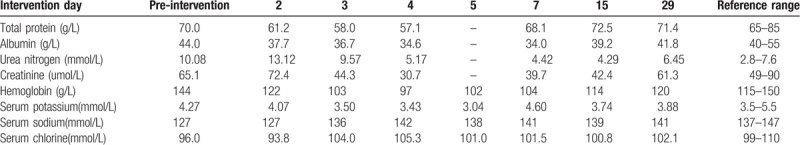
Change in laboratory data from nutritional intervention to discharge.

On January 17th, 2018, the patient was transferred to Department of Rehabilitation Medicine for further rehabilitation training, and her muscle strength of limbs increased gradually. As her swallowing function gradually recovered, the nasogastric tube was removed and the oral diet was started. The patient finally achieved the goal of rehabilitation and was discharged on February 9th, 2018.

The patient provided informed consent for publication of the case report.

## 3 Discussion

We report a case of WE in a patient with cerebral infarction, which is uncommon. In this case, the patient was diagnosed with anxiety and depression, which resulted in nausea, vomiting and poor appetite, it was defined as psychogenic nausea and vomiting and psychogenic poor appetite. Previous studies have shown that lesions in the right temporal lobe of stroke patients can cause emotional changes and are related to anxiety and depression.^[9]^ Thus, in this case, the patient's temporal lobe infarction caused anxiety and depression, which further led to nausea, vomiting and poor appetite. These factors ultimately resulted in malnutrition and thiamine deficiency. The early stages of thiamine deficiency can inversely cause gastrointestinal symptoms such as slow gastric emptying, nausea and vomiting.^[10]^ Thus a vicious circle was formed. Her weight spiraled down. Attributing to sharp fall in her weight, the patient was diagnosed with malnutrition. Previous studies have shown that rapid weight loss and malnutrition can result in WE, and the occurrence of WE is directly linked to rapid weight loss.^[11,12]^

Dysphagia is a commonly complaint after strokes. A systematic review found that the reported incidence of dysphagia was 64% to 78% with instrumental testing in stroke patients.^[13]^ Dysphagia can cause severe complications of malnutrition and/or dehydration in up to 25% of stroke patients.^[14]^ Therefore, malnutrition in patients with cerebral infarction should be given full attention. When patients are diagnosed with malnutrition, a reasonable and personalized nutrition therapy should be administered by a professional clinical nutritionist to correct malnutrition in a timely manner. In this way, somatic complications caused by malnutrition, such as WE, can be avoided.

WE is diagnosed primarily by the typical triad syndrome,^[15]^ but only 16% of WE patients had the typical triad syndrome and 19% had no documented clinical signs.^[3]^ MRI typically show an high T2 signal in the paraventricular regions of the thalamus, the hypothalamus, mamillary bodies, the periaqueductal region, the floor of the fourth ventricle and midline cerebellum, which was consistent with our report.^[16]^ The sensitivity and specificity of MRI for diagnosing WE are 53% and 93% respectively, the positive predictive value of MRI is 89%.^[17]^ The high specificity can be used to rule out other intracranial pathologies, but the sensitivity is low. In patients with WE, positive MRI results are associated with altered mental status, but are not related to the other two of the typical triads.^[11]^ computerized tomography is not useful for diagnosing WE.^[17]^ There are the operational criteria proposed by Caine for the diagnosis of WE, which require 2 of the following 4 signs:

(1) dietary deficiencies,
(2) oculomotor abnormalities,
(3) cerebellar dysfunction, and
(4) either an altered mental state or mild memory impairment.^[18]^

In a previous systematic review, the use of the criteria in the diagnosis of WE was found that Caine's criteria had a higher sensitivity than the typical triad.^[2]^ In this case report, the patient had poor appetite because of the onset of cerebral infarction approximately 1 month before admission. At the time of admission, she had lost 15 kg of body weight (weight loss rate was 23.1%), and was diagnosed with malnutrition after a nutritional assessment, which proved that the condition of dietary deficiency was existed. In addition, the patient manifested as tiny right horizontal nystagmus on admission. Therefore, according to Caine's criteria, she should be diagnosed with WE at this point. Her delayed diagnosis might result from the clinicians’ low awareness of these criteria. Achieving the early diagnosis of WE remains a challenge for clinicians, and the use of Caine's criteria could increase the early diagnosis of WE. Besides, the diagnosis of WE may be masked by other diseases because its symptoms are common to many illness.^[19]^ Cerebral infarction can cause the instability of gait, nystagmus (ie, basilar artery occlusion), disturbance of consciousness, which can also occur in patients with WE. These symptoms cause cerebral infarction to be difficult to distinguish from Wernicke's encephalopathy. WE is a brain disorder with a clear response to thiamine treatment, but cerebral infarction does not recover in the short term based on nutrient supplementation. Thiamine therapy can rapidly improve ataxia, ophthalmoplegia and nystagmus, as well as confusional state in patients with WE.^[20]^ It has been reported that eye movement disorders can be improved after a few hours of treatment. The recovery of ataxia occurs after a few days. The changes in mental state tend to improve after 2 to 3 weeks of treatment.^[16]^ This aspect can be used as a good identification point between WE and cerebral infarction. Caine's criteria use the broadest definition of clinical symptoms rather than the narrower classic triad, and emphasize the importance of malnutrition.^[19]^ For patients with cerebral infarction, if there are dietary deficiencies or malnutrition, preventive thiamine supplementation should be administered to prevent further progression of the disease. And the level of suspicion for WE should be high in cerebral infarction patients whose clinical condition may lead to thiamine deficiency.

Thiamine is an essential water-soluble vitamin. In addition, thiamine is an essential coenzyme for numerous biochemical pathways of the nervous system. The Chinese recommended nutrient intake of dietary thiamine in adults is 1.4 mg/d for men and 1.2 mg/d for women, and increase in children, pregnancy, lactation, malignant diseases, and the high caloric or high carbohydrate diet.^[16]^ The body is capable of storing 30 to 50 mg of thiamine.^[21]^ Because the body's storage of thiamine are adequate for up to 18 days.^[22]^ When a healthy individual takes imbalanced nutrition lasting 2 to 3 weeks, it may result in thiamine depletion and WE.^[16]^ Vomiting, intravenous glucose without thiamine and parenteral nutrition are predisposing factors of WE.^[23]^ Due to prolonged emesis and poor appetite, the body of the patient was depleted of thiamine. The clinician gave her intravenous glucose without thiamine and parenteral nutrition, which aggravated her thiamine deficiency.

WE is a rare neuropsychiatric disorder with specific symptomatic treatment, and thiamine repletion is the only treatment for WE. Intravenous treatment with either 100 or 200 mg thiamine has cured WE in non-alcoholics based on many case reports.^[4]^ According to the European Federation of Neurological Societies and the Royal College of Physicians, it is recommended to administer 500 mg of thiamine intravenously three times daily in alcoholic patients until symptoms of acute WE resolve.^[4,24]^ The EFNS also recommends that suspected or manifest WE should be given 200 mg of thiamine 3 times daily and preferably by intravenous rather than intramuscular route until symptoms and signs are not further develop.^[4]^ Thiamine should be administered before any carbohydrate, and a balanced diet should be established immediately after thiamine.^[4]^ In this case, thiamine supplementation and balanced nutritional therapy were given immediately after the diagnosis of WE.

If the diagnosis and treatment are delayed, WE will progress to chronic Korsakoff syndrome, which is characterized by amnestic disorder and confabulation.^[19]^ In order to avoid unnecessary delay, clinicians should administer thiamine at the earliest awareness of risk factors for thiamine deficiency or suspicion of WE.^[2]^

In summary, patients with cerebral infarction often have the complications of malnutrition, which may result in nutrient deficiency-related neurological complications, such as WE. Therefore, it is important to pay close attention to the nutritional status of patients with cerebral infarction, and correct the malnutrition reasonably. There were delays in diagnosis and treatment of WE in this case, suggesting that we could use Caine's criteria for early diagnosis. Besides, early recognition and differential diagnosis of WE in patients with infarction-related malnutrition are necessary, and early treatment of replete thiamine supplementation and nutrition support therapy can reduce the risk of WE and improve the prognosis.

## Acknowledgments

We would like to thank the patient for his informed consent and contribution to the publication of this case report. We also thank the doctors of Department of Geriatrics and Department of Nutrition for their advice and help.

## Author contributions

**Conceptualization:** Ping Sun, Xiaojiao Lian.

**Supervision:** Xiaojiao Lian, Meng Wu, Haixia Fan, Yi Zhang, Ping Sun.

**Writing – original draft:** Xiaojiao Lian.

**Writing – review & editing:** Xiaojiao Lian, Meng Wu, Ping Sun.
